# Cannabinoid hyperemesis and the cyclic vomiting syndrome in adults: recognition, diagnosis, acute and long-term treatment

**DOI:** 10.3205/000247

**Published:** 2017-03-21

**Authors:** Christian G. Blumentrath, Boris Dohrmann, Nils Ewald

**Affiliations:** 1 Department of Emergency Medicine, General Hospital Luebbecke-Rahden, Rahden, Germany; 2 Department of Internal Medicine, General Hospital Luebbecke-Rahden, Rahden, Germany; 3 Justus-Liebig-University Giessen, Germany

**Keywords:** nausea, vomiting, abdominal pain, hot showering/hot bathing, cannabis, periodic vomiting, cannabinoid hyperemesis, cyclic vomiting in adults

## Abstract

The cannabinoid hyperemesis syndrome (CHS) and the cyclic vomiting syndrome in adults (CVS) are both characterized by recurrent episodes of heavy nausea, vomiting and frequently abdominal pain. Both syndromes are barely known among physicians. Literature is inconsistent concerning clinical features which enable differentiation between CVS and CHS.

We performed a literature review using the LIVIVO search portal for life sciences to develop a pragmatic approach towards these two syndromes. Our findings indicate that complete and persistent resolution of all symptoms of the disease following cannabis cessation is the only reliable criterion applicable to distinguish CHS from CVS. Psychiatric comorbidities (e.g. panic attacks, depression), history of migraine attacks and rapid gastric emptying may serve as supportive criteria for the diagnosis of CVS. Compulsive bathing behaviour, a clinical observation previously attributed only to CHS patients is equally present in CVS patients.

Long-term follow-up is essential in order to clearly separate CHS from CVS. However, long-term follow-up of CVS and CHS cases is seldom. We provide a standard operating procedure applicable to a broad spectrum of health care facilities which addresses the major issues of CVS and CHS: awareness, diagnosis, treatment, and follow-up.

## Zusammenfassung

Die typischen Symptome sowohl des Cannabis-Hyperemesis-Syndroms (CHS) als auch des Syndroms des zyklischen Erbrechens beim Erwachsenen (CVS) sind wiederholt auftretende, heftige Übelkeit, Erbrechen und häufig auch Bauchschmerzen. Für beide Syndrome gilt, dass sie auch in Medizinerkreisen weitgehend unbekannte Erkrankungen darstellen. Die Literatur ist uneinheitlich bezüglich der klinischer Symptome, die eine Unterscheidung der beiden Syndrome ermöglichen.

Wir haben eine pragmatische Herangehensweise an diese beiden Syndrome entwickelt, die auf den Ergebnissen einer Literaturrecherche über das Such-Portal für Lebenswissenschaften LIVIVO basiert. Unsere Ergebnisse zeigen, dass einzig das dauerhafte und vollständige Verschwinden aller Symptome nach Beenden des Cannabiskonsums geeignet ist, diese beiden Syndrome zuverlässig voneinander zu unterscheiden. Psychiatrische Begleit- oder Vorerkrankungen (z.B. Panikattacken, Depressionen), Migräneanfälle oder auch eine beschleunigte Magenentleerung können bestenfalls als unterstützende Diagnosekriterien herangezogen werden. Das zwanghafte heiße Duschen, das ursprünglich dem Cannabis-Hyperemesis-Syndrom zugeschrieben wurde, kommt bei beiden Erkrankungen ähnlich häufig vor.

Daher ist eine lange Nachverfolgungszeit der Patienten notwendig um sich auf eine der beiden Diagnosen festlegen zu können. Leider ist dies in den bisher veröffentlichten Fallberichten und Fallserien selten gewesen. Wir haben eine Verfahrensanweisung entwickelt, die in vielen unterschiedlichen klinischen Settings anwendbar ist und die wesentlichen Punkte bezüglich beider Syndrome abdeckt: Bekanntheitsgrad, Diagnostik, Behandlung und Nachverfolgung.

## Introduction

**Figure 1:**
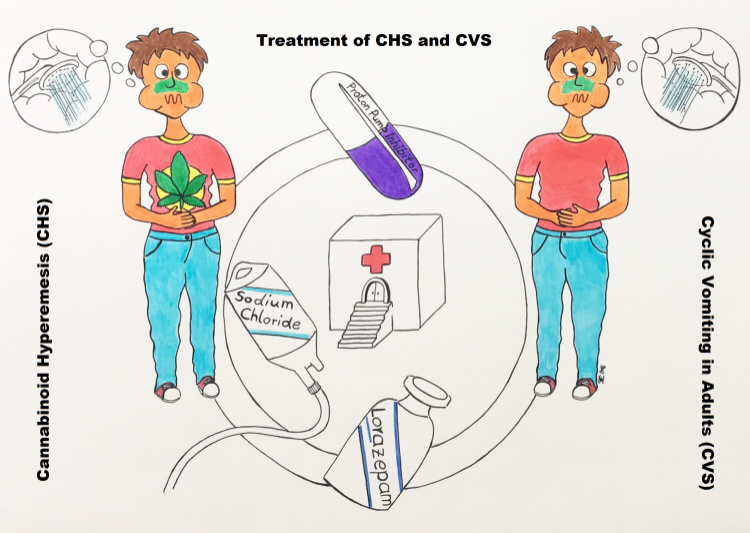
Patients present with heavy nausea, vomiting and frequently abdominal pain. Approximately 50% of the patients display compulsory bathing behaviour as hot showering results in symptoms relief. Chronic marihuana abuse is a prerequisite for suspicion of CHS. Prompt and adequate treatment of an episode of vomiting shortens the recovery phase and prolongs the inter-episodic phase of comparative wellbeing. Treatment of the acute phase consists of intravenous application of lorazepam, proton pump inhibitors, and sodium chloride solution. Detailed information on diagnostic criteria and treatment recommendations: Tables 1 and 3.

Medical literature recognises two syndromes, the cyclic vomiting syndrome in adults (CVS) and the cannabinoid hyperemesis syndrome (CHS) which are both characterised by recurrent episodes of heavy nausea, vomiting (see Figure 1 (Fig. 1)) and comparative well-being between the episodes [1], [2]. A prerequisite for the diagnosis of both syndromes is absence of an obvious organic cause for the displayed symptoms [1], [2].

CVS in adults displays 4 phases [1]. During the inter-episodic phase, patients are relatively free of symptoms [1]. Triggers, e.g. noxious stress, pleasant excitement, infections or menstrual periods may lead to transition into the prodromal phase [1]. The prodromal phase begins when the patient senses the approach of an episode and is characterized by nausea which still allows oral medication [1]. If not adequately treated, patients enter the episode of vomiting which lasts from <12 hours up to >7 days [1]. Subsequently, the recovery phase begins with the cessation of vomiting and ends when hunger and oral intake return to normal [1]. 

CHS patients have a long prodromal phase (up to several years) which is characterised by nausea, abdominal pain, and fear of vomiting while the patients maintain normal eating patterns [2]. During the hyperemesis phase, patients experience heavy nausea, vomiting, and abdominal pain [2]. The recovery phase begins with cessation of cannabis use and can last for days up to months [2]. Return to cannabis use inevitably leads to recurrence [2]. 

During the emetic phase, intravenous lorazepam, proton pump inhibitors, and fluid substitution are generally recommended in both syndromes [1], [2]. Conventional antiemetic and analgesic treatment is insufficient [1], [2]. Consequent cannabis cessation leads to complete and persistent resolution of symptoms in CHS patients [2]. CVS patients should receive preventive treatments, e.g. propranolol, amitriptyline or migraine medications and medication to abort the emetic phase in case of prodromal symptoms, e.g. ondansetron, lorazepam, oxycodone.

Literature is inconsistent concerning clinical features which allow to differentiate CVS from CHS [1], [2], [3], [4], [5], [6], [7], [8]. Both syndromes are largely unknown. Therefore, the available data relies on case reports and case series [1], [2], [3], [4], [5], [6], [7], [8]. Patients suffering from either syndrome frequently have a long medical record and undergo avoidable, potentially harmful diagnostics (endoscopic examination, computed tomography, X-rays of the abdomen etc.) and therapeutic procedures (e.g. cholecystectomy, appendectomy) before diagnosis is established [1], [2]. 

The aim of this review was to compare the patterns of disease of CVS and CHS. The similarities of both syndromes indicated the need for development of a pragmatic approach towards both CVS and CHS, applicable to a broad range of clinical settings.

## Materials and methods

On September 17, 2016, we performed a literature search using the LIVIVO search portal for life sciences (https://www.livivo.de/) which accesses several databases (for detailed information access website). Search terms used were: “cyclic vomiting”, “cannabinoid hyperemesis”, “hot showering, nausea, vomiting” without language restrictions. We screened titles and, where available, abstracts of all records identified for potential information concerning either of the syndromes. Additionally, we screened the references of all articles on the subject and added papers which were not previously detected.

## Epidemiology of CVS and CHS

Both syndromes are largely unknown among physicians [1], [2]. Data substantially relies on case reports and case series [1], [2], [3], [4], [5], [6], [7], [8]. Reliable prevalence data does not exist for both syndromes [1], [2], [3], [4], [5], [6], [7], [8]. 

The cyclic vomiting syndrome in infants and children has an estimated prevalence of 0.04–2% [3], [4]. Prevalence of CVS in adults is suspected to be significantly lower [3], [4]. During the last decades, greater awareness has led to an increasing number of case reports and case series, which could indicate a high unreported number of undiagnosed CVS cases [4].

Since Allen et al. raised the hypothesis of cannabinoid hyperemesis [5], the number of cases and case series reporting on the topic has steadily grown [6]. Darmani suggested that there is growing evidence that CHS is not as rare among chronic cannabis abusers as initially estimated [6]. In Colorado, the number of admissions to the department of emergency medicine (ED) due to CHS has nearly doubled since legalisation of cannabis use (41 per 113,262 ED visits before, 87 per 125,095 ED visits after legalisation), which supports this hypothesis [7]. 

## Recognition of CVS and CHS

Awareness of CVS and/or CHS and detailed history of the patient is the key to suspicion and diagnosis in patients presenting with nausea and vomiting (Figure 1 (Fig. 1)). First symptoms occur at the age of 22±5 years. It takes about 10 years until definitive diagnosis is established. 

The typical CVS/CHS patient is a middle-aged, Caucasian male adult (average age at diagnosis: 35 years, range 16 years – 65 years; approximately 80% of patients are Caucasian; male/female ratio: 3:2–7:3) [1], [8]. These patients frequently have a long medical record and underwent multiple diagnostic measures and even surgical interventions without any identification of an organic cause of or cure from symptoms [1], [2]. 

Typically, CVS and CHS patients report recurrent (cyclic) episodes of heavy nausea and vomiting, frequently accompanied by (severe) abdominal pain [1], [8]. Low grade fever, headache, loose stools and even diarrhoea may be present on admission [1], [4], [8]. The average duration of a vomiting episode ranges from 3 to 4 days (variable from a few hours to more than one week) [1], [8]. Duration of the recovery phase is extremely variable and strongly depends on adequate treatment [1], [8]. During phases of comparative wellness between episodes, which vary largely in length between weeks or several months, patients are free of symptoms or report occasional nausea, abdominal pain and even vomiting [1], [2].

A unique feature of CVS and CHS is symptoms relief by hot showering or bathing, reported by approximately 60% of patients [1], [2], [9], [10]. About 60% of patients complain about severe abdominal pain which is mostly located to the periumbilical or epigastric region [1], [2]. During episodes, patients may display (psycho-)vegetative symptoms, e.g. sweating, irritability or agitation [1], [5], [8]. Even before dehydration, polydipsia can be present [1], [5]. Vomiting from an empty stomach seems to be more painful than vomiting from a water-filled stomach which results in excessive oral intake of water leading to waterish-foamy vomits in numerous patients [1]. However, all other forms of vomit were reported from different cases [1], [11]. 

The importance of early case detection lies in avoidance of long waiting time and unnecessary and potentially harmful diagnostic measures on the one hand and the administration of adequate treatment on the other hand. Immediate and adequate treatment shortens the recovery phase and lengthens the durations of the inter-emetic phase [1], [2]. 

Standard operating procedures for detection, diagnosis, treatment and follow-up of patients potentially suffering from CVS or CHS provide a useful tool to increase awareness and help clinicians to address these patients adequately (see Attachment 1) [12]. 

## Diagnosis of CVS and CHS

Taking a detailed history of the patient is the key to diagnosis. Applying our criteria for the diagnosis of CVS and CHS (Table 1 (Tab. 1)) is useful to corroborate suspicion. Differential diagnostic considerations of nausea and vomiting encompass diagnoses from various clinical disciplines (Table 2 (Tab. 2)). Among these, diagnosis of CVS and CHS is rare. 

Upon clinical examination, most patients do not reveal findings indicating an organic cause of the disease [1], [8]. However, low grade fever, signs of dehydration, and abdominal tenderness were found in some patients [1], [2], [3], [4], [5], [6], [7], [8], [9], [10], [11], [13], [14], [15]. There should be no indication of a neurological cause of the displayed symptoms [1], [2], [3], [4], [5], [6], [7], [8], [9], [10], [11], [13], [14], [15].

Laboratory examination may reveal leucocytosis, electrolyte imbalances, elevated amylase levels and, rarely, acute renal failure [1], [2], [3], [4], [5], [6], [7], [8], [9], [10], [11], [13], [14]. Calcium levels, C-reactive protein levels, lipase, liver enzymes, thyroid parameters, transglutaminase and gliadin antibodies are generally normal [1], [2].

Abdominal ultrasound, oesophago-gastro-jejunoscopy including biopsy and gastric emptying speed examination should be performed in all cases of suspicion of CVS and CHS [1], [2]. Usually, these diagnostic features reveal normal findings [1], [2]. However, Mallory-Weiss lesions, oesophagitis and gastritis may be detected in some cases of both syndromes [1], [2]. The use of (contrast enhanced) computed tomography and magnetic resonance imaging in cases matching all essential (based on history of the patient, clinical, laboratory, and ultrasound findings) and at least 3 major criteria for diagnosis of CVS and CHS (Table 1 (Tab. 1)) should be avoided where possible [1], [2]. Rapid gastric emptying indicates CVS whereas delayed gastric emptying is more frequently found in CHS patients [13], [14].

## Treatment of CVS and CHS

Delay of adequate treatment of CVS and CHS patients results in prolonged recovery time and shortened inter-episodic phases of comparative wellness [1], [2]. Table 3 (Tab. 3) illustrates therapeutic regimes of CVS and CHS.

Patients in the acute phase of either syndrome do not respond adequately to conventional treatment (e.g. metamizole, metoclopramide, alizaprid, dimenhydrinate, ondansetron) of nausea, vomiting and abdominal pain [1], [2]. Relief of syndromes can be achieved by intravenous administration of lorazepam, alprazolam and, as second line treatment, haloperidol [1], [2]. Administration of proton pump inhibitors and intravenous sodium chloride 0.9% (1–2 l bolus followed by 150–200 ml/h for 24–48 hours) until cessation of vomiting is generally recommended [1], [2]. Patients should be provided access to hot showering or bathing for symptoms relief [4].

The main treatment goal of patients who report chronic marijuana abuse is cannabis cessation [2], [5]. In CHS patients, cure can be achieved by cessation of cannabis consumption alone [2], [5], [6]. Return to cannabis abuse inevitably leads to relapse [2], [5], [6]. Haloperidol was reported to be effective in CHS patients who refuse cannabis cessation [10].

In patients suffering from CVS, there is consensus that application of preventive medication and medication capable of aborting an episode reduces the intensity and frequency of cycles [1], [3], [4]. Amitriptyline, propranolol, sumatriptane are recommended preventive medications [1], [3], [4]. Metoclopramide, ondansetron, lorazepam or oxycodone, ideally with application at the onset of prodromal symptoms, can abort an episode [1], [3], [4]. Psychosocial care is of additional benefit [1], [3], [4].

In patients who refuse cessation of cannabis use and especially in patients who do not sufficiently respond to cannabis use cessation alone, adopting the therapeutic regime of CVS might be beneficial. But there is no data supporting a potential benefit of applying the therapeutic strategy of CVS to CHS in these patients.

## Follow-up of CVS and CHS

Reliable long-term follow up data (minimum follow up time: 12 months) of patients suffering from CVS or CHS is sparse [1], [2], [3], [4], [5], [6], [7], [8], [9], [10], [11], [13], [14], [15]. Differentiation between CVS and CHS is simple in patients who do not practise chronic marijuana abuse [1], [2]. Distinguishing between CVS from CHS in patients who practise chronic marijuana abuse can be extremely difficult [1], [3], [5], [9], [11]. 

Follow-up involving these patients pursues five main objectives:

1. Evaluation of complete and permanent resolution of symptoms due to cannabis cessation alone.
2. Evaluation of the effectiveness of therapeutic strategies for CVS when applied to CHS patients who refuse cannabis cessation.
3. Evaluation of the efficacy of therapeutic strategies for CVS patients in patients who do not fully respond to cannabis cessation.
4. Providing access to physicians familiar with CVS and CHS to patients suffering from either syndrome.
5. Collection of data helpful to evaluate therapeutic strategies in CVS and CHS patients [1], [2], [3], [4], [5], [6], [7], [8], [9], [10], [11], [13], [14], [15].

Patients who fully respond to cannabis cessation alone for a minimum period of 12 months after complete resolution of symptoms are likely to suffer from CHS [2], [5], [6], [8]. Diagnosis in patients who cease cannabis abuse and continue to have symptoms is probably CVS. Patients who continue cannabis abuse but benefit from therapeutic regimes available for CVS patients may be diagnosed CVS [1], [2]. The occurence of patients who do not cease cannabis abuse and continue to have symptoms despite treatment alike CVS patients support the hypothesis that cannabis cessation is the only available treatment for CHS [2]. 

## Discussion

From a practitioner’s point of view, the major issue of CVS and/or CHS is the limited awareness among physicians which consequently results in diagnostic failure and inadequate treatment [1], [2]. Displaying educational material in poster format in departments of emergency medicine may increase awareness among physicians and patients. We encourage our readers to share Figure 1 (Fig. 1) in social media. Standard operating procedures (SOP) for detection, diagnosis, treatment and follow up of patients suffering from CVS or CHS (see Attachment 1) provide a valuable method to address this problem in hospitals at low costs [12]. Not only will it be more likely that patients will be recognized as suffering from CVS/CHS, they are also more likely to receive adequate treatment [12]. 

Diagnosis of CVS/CHS is an interdisciplinary approach and provides a challenge to all disciplines involved [1], [2], [3], [4], [5], [6], [7], [8], [9], [10], [11], [13], [14], [15]. Differential diagnosis is extremely broad (Table 2 (Tab. 2)). A detailed history of the patient and a thorough clinical examination may justify corroborated suspicion [1], [2]. Laboratory examination, abdominal ultrasound, oesophago-gastro-jejunoscopy and gastric emptying speed analysis should be performed in every suspected case. In a great number of patients, history of the patient, clinical examination, laboratory examination and abdominal ultrasound is sufficient for diagnosis of CVS or CHS. Other cases require expert consultation for diagnosis. In some (atypical) cases, findings may indicate an organic cause which then requires extended examination including radiographic imaging, e.g. computed tomography or magnetic resonance imaging. Consultation of a psychiatric expert can be necessary to rule out psychogenic vomiting, eating disorders and psychiatric comorbidities. However, applying our criteria for the diagnosis of CVS and CHS (Table 1 (Tab. 1)) to a patient’s history of disease and clinical presentation will increase the likelihood of correct diagnosis significantly and may help avoid unnecessary diagnostic features [1], [2], [3], [4], [5], [6], [7], [8], [9], [10], [11], [13], [14], [15]. 

Evidence supporting the hypothesis of cannabinoid hyperemesis is weak. The concept of CHS is based on the hypothesis of a paradoxical effect of cannabis (in long-term abuse) due to pharmacodynamical and pharmacokinetic variations in susceptible individuals [2], [5], [6]. Although the potential pathophysiologic mechanisms (of CVS and CHS) remain obscure, animal studies and findings from basic research support the hypothesis of a paradoxical effect of cannabis in long-term abuse [4], [6]. While paradoxical effects of cannabinoids are well known concerning emotions (e.g. relaxing effects vs. paranoia; euphoria vs. dysphoria), a potential emetic effect of cannabinoids is relatively unknown [6], [15]. However, there is evidence that in chronic marijuana users suffering from CVS, marijuana could induce a cycle in about 5% of all cases [4]. Chronic marijuana abuse is an insufficient criterion to distinguish CHS from CVS [1], [9], [11]. It is extremely difficult to separate CHS from CVS in patients who use cannabis on a regular basis. Although some clinical findings (psychiatric comorbidities, migraine, rapid gastric emptying) may make an indication towards one or the other, these features are at best of supportive nature (Table 1 (Tab. 1)). The characteristics of the prodromal phase in CHS patients seems to be significantly different from CVS [1], [2], [3], [4], [5], [6], [7], [8]. Fleisher et al. described the phenomenon of coalescence of episodes over time in CVS patients [1]. Coalescence of episodes over time describes a worsening of symptoms and an abbreviation of inter-episodic phases in CVS patients who are not adequately treated [1]. It is possible that the prodromal phase in CHS patients is equivalent to the time before coalescence of episodes over time in CVS patients. 

Complete and persistent (at least 12 months) resolution of all symptoms following cannabis cessation alone is the best existing clinical evidence supporting the hypothesis of cannabinoid hyperemesis [1], [5]. However, the existing evidence is subject to bias:

1. Long-term follow-up data is sparse.
2. Patients willing to cease cannabis abuse frequently received medical (e.g. lorazepam) and psychosocial support from the physicians treating them [5]. Adequate treatment of acute hyperemesis in CVS patients, preventive medication and psychosocial supportive care, significantly improves symptoms, lengthens the inter-emetic phase of well-being and even leads to cure in some cases [1].
3. Case studies and case series reporting on patients who refused cannabis cessation and continued to have symptoms do not support the hypothesis of CHS. Frequently, these patients were only treated during the hyper-emetic episode and did not receive any further treatment as there is/was consensus that cannabis cessation is the only treatment available for CHS [2]. However, cure following treatment with haloperidol was reported in patients who continued marijuana abuse [10].
4. In a great number of cases published, diagnosis of CHS was based on improvement of symptoms following an episode and cannabis cessation [5]. Improvement of symptoms during an episode and during the recovery phase is part of both syndromes, CVS and CHS [1], [2].

Data concerning prevalence and long-term follow-up of CVS and CHS is extremely sparse [1], [2], [3], [4], [5], [6], [7], [8], [9], [10], [11], [13], [14], [15]. Applying our standard operating procedure to numerous clinical institutions may improve detection of cases, treatment of these patients and may help generate reliable data concerning long-term follow up and prevalence of CVS/CHS within these institutions. Integrating CHS into studies evaluating chronic marijuana abuse can give an idea of prevalence of CHS within this group.

## Conclusion

We provide a reliable and feasible clinical approach towards two clinically extremely similar syndromes (CHS and CVS). This pragmatic approach encompasses the major issues of both syndromes: awareness, recognition and adequate diagnostic measures, treatment and follow-up. Additionally, this approach can generate data which is required to better understand and treat CVS and CHS. 

## Notes

### Competing interests

The authors declare that they have no competing interests.

### Financial disclosure

The authors received no funding for this analysis. 

### Acknowledgement

Julia Edinger was responsible for the artistic realisation of Figure 1 (Fig. 1) and kindly provided her work for publication in this article. The authors thank the team of doctors and nurses of the different departments of Muehlenkreiskliniken, General Hospital Luebbecke-Rahden. CGB thanks Dr. med Barbara Hogan for English language grammar and spell-check of this article and especially for her support during a long and fruitful cooperation.

### Authors’ contributions 

CGB conceived the idea for this article, performed literature search and drafted a first version of the manuscript. All authors reviewed the results of the literature search and contributed substantially to the final version of the manuscript. All authors read and approved the final manuscript.

## Supplementary Material

Standard Operating Procedure: Detection, evaluation, treatment and follow up of patients possibly suffering from the Cyclic Vomiting Syndrome in adults/Cannabinoid Hyperemesis Syndrome

## Figures and Tables

**Table 1:**
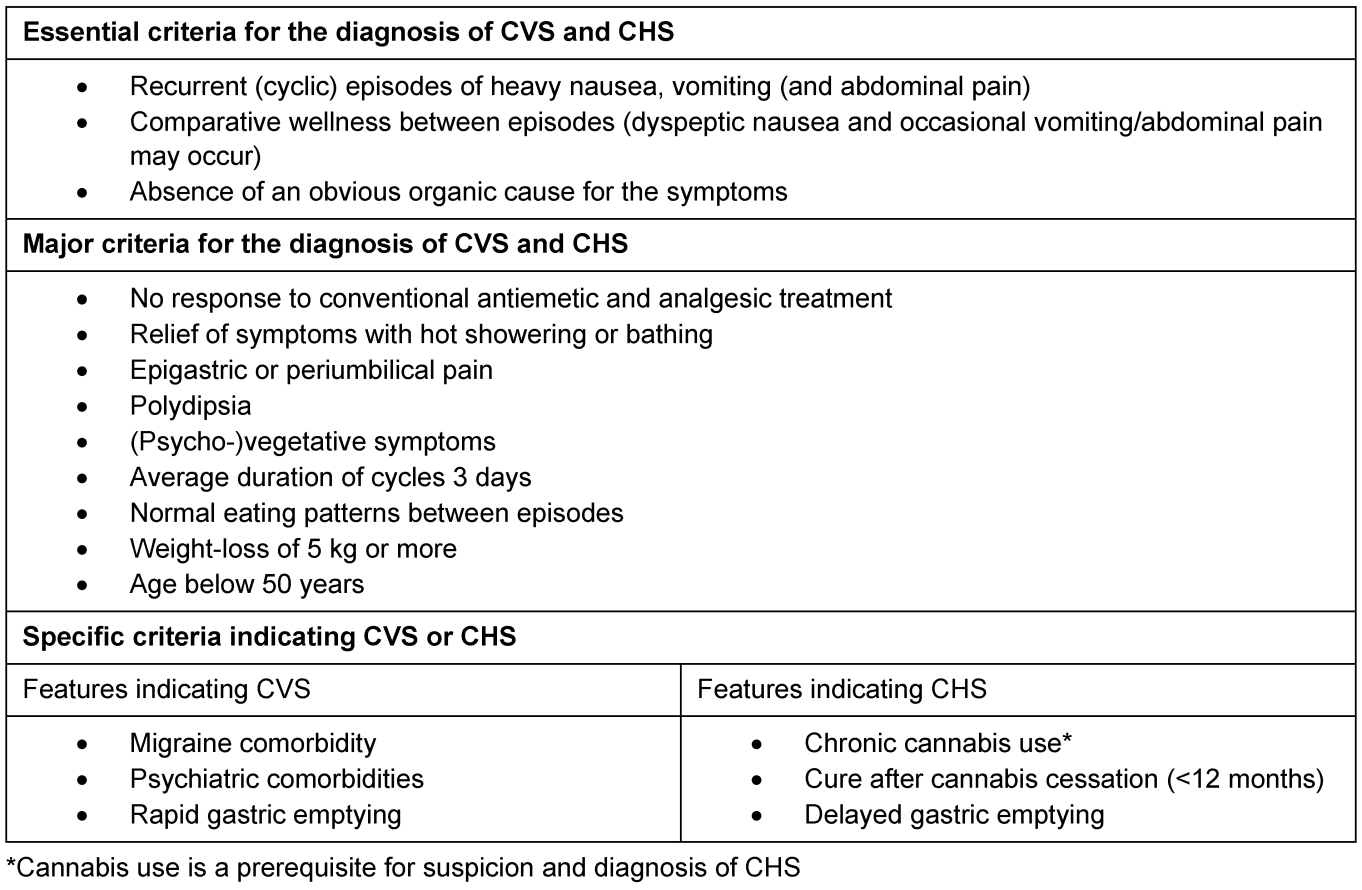
Criteria for the diagnosis of CVS and CHS

**Table 2:**
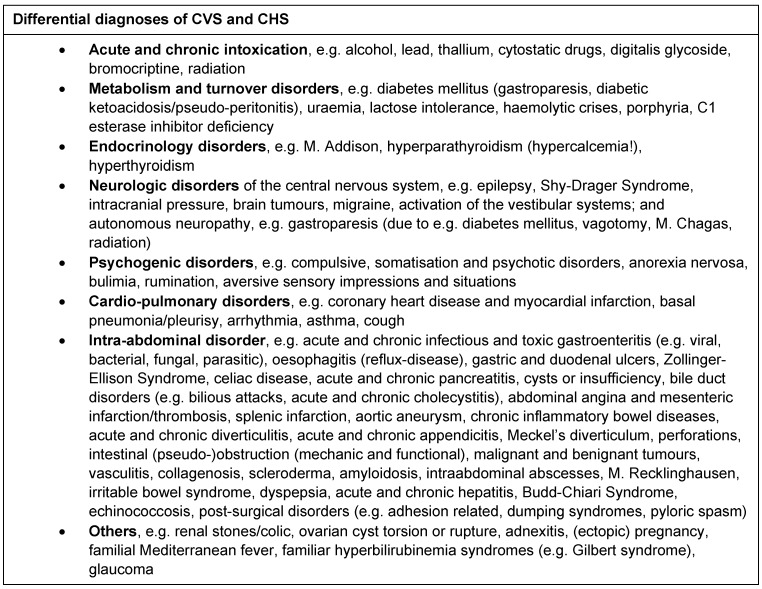
Differential diagnoses of CVS and CHS

**Table 3:**
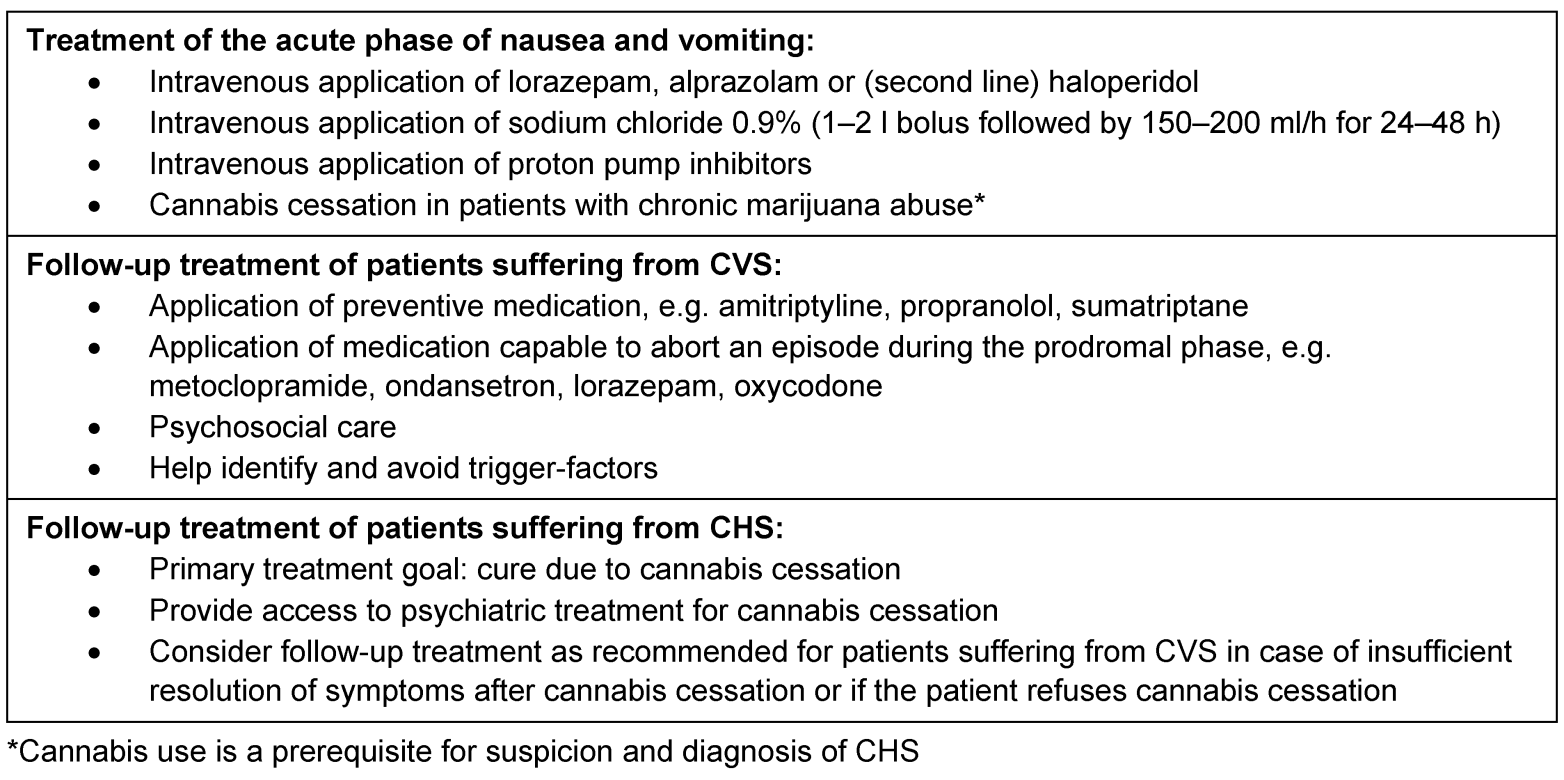
Treatment
