# Supplementary material for: Cannabinoid hyperemesis and the cyclic vomiting syndrome in adults: recognition, diagnosis, acute and long-term treatment
Source: Ger Med Sci. 2017 Mar 21;15:Doc06. doi: 10.3205/000247 (PMC5360975; doi:10.3205/000247)
Supplement: Standard Operating Procedure: Detection, evaluation, treatment and follow up of patients possibly suffering from the Cyclic Vomiting Syndrome in adults/Cannabinoid Hyperemesis Syndrome [file GMS-15-06-s-001.pdf]

Standard Operating Procedure:
Detection, evaluation, treatment and follow up of patients possibly suffering from the Cyclic Vomiting Syndrome in adults/Cannabinoid Hyperemesis Syndrome

Background

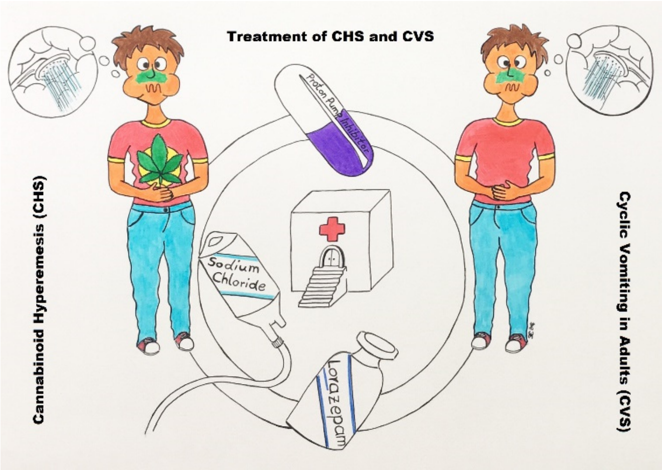

The Cyclic Vomiting Syndrome in adults (CVS) and the Cannabinoid Hyperemesis Syndrome (CHS) are characterised by recurrent episodes of heavy nausea, overwhelming vomiting and abdominal pain without an obvious organic cause of the symptoms. The major difference is that in CHS patients the suspected cause of disease is chronic cannabis abuse (at least one a week during the last few months). Both syndromes are largely unknown which frequently leads to excessive diagnostic measures, multiple hospital admissions and inadequate treatment. Data concerning both syndromes is sparse. The purpose of this standard operating procedure (SOP) is to identify potential cases, avoid unnecessary diagnostic measures, and provide adequate treatment and follow up of patients suffering from either syndrome.

Literature/Source

The recommendations of this SOP are based on:

- Sun S, Zimmermann AE. Cannabinoid hyperemesis syndrome. Hosp Pharm. 2013 Sep;48(8):650-5. DOI: 10.1310/hpj4808-650
- Fleisher DR, Gornowicz B, Adams K, Burch R, Feldman EJ. Cyclic Vomiting Syndrome in 41 adults: the illness, the patients, and problems of management. BMC Med. 2005 Dec;3:20. DOI: 10.1186/1741-7015-3-20

Standard Operating Procedure:
Detection, evaluation, treatment and follow up of patients possibly suffering from the Cyclic Vomiting Syndrome in adults/Cannabinoid Hyperemesis Syndrome

Criteria for Diagnosis of CVS and CHS

Essential criteria for the diagnosis of CVS and CHS

- Recurrent (cyclic) episodes of heavy nausea, vomiting (and abdominal pain)
- Comparative wellness between episodes (dyspeptic nausea and occasional vomiting/abdominal pain may occur)
- Absence of an obvious organic cause for the symptoms

↓

First view*

Consider triage results, take brief history of the patient and perform a preliminary physical examination. Determine preliminary blood/urine analysis and further evaluation procedures. Initiate an intravenous line and determine the preliminary treatment (fluid therapy, analgesia (intravenous paracetamol or metamizole) and antiemetic (alizaprid, metoclopramid, demenhydrinate) treatment). Do the results of your preliminary examination possibly match the criteria for diagnosis of CVS/CHS?

Yes ↓   No ↓

Indicate your suspicion to the attending physician in the ED.
Proceed to page 3

*According to: Hogan B, Rasche C, von Reinersdorff AB. The First View Concept: introduction of industrial flow techniques into emergency medicine organization. Eur J Emerg Med. 2012 Jun;19(3):136-9. DOI: 10.1097/MEJ.0b013e32834bbd93

Standard Operating Procedure:
Detection, evaluation, treatment and follow up of patients possibly suffering from the Cyclic Vomiting Syndrome in adults/Cannabinoid Hyperemesis Syndrome

Extended evaluation

Take detailed history of the patient and perform a thorough clinical examination. Perform abdominal ultrasound examination and extend laboratory diagnostic (calcium-level?) if necessary. Consider previous suspicions, laboratory results, and response to previously initiated treatment. Consult with specialists if indicated. Does your evaluation indicate any organic cause of the displayed symptoms?

Yes

Initiate necessary diagnostic procedures and treatment matching your suspicion; return to SOP if your evaluation does reveal any organic cause of the displayed symptoms.

No

Treatment of vomiting episodes

Start treatment of the emetic episode of CHS and CVS: intravenous sodium chloride 0.9% (1–2 l bolus followed by 150–200 ml/h for 24–48 h) and proton pump inhibitors; treat electrolyte imbalances if indicated; intravenous application of lorazepam or alprazolam (haloperidol); in case of chronic marijuana use explain the need for cannabis cessation.

Approach

Inform the ward and the physician responsible for further diagnostics and treatment of the patient about your suspicion; for further treatment and follow-up proceed to page 4

Standard Operating Procedure:
Detection, evaluation, treatment and follow up of patients possibly suffering from the Cyclic Vomiting Syndrome in adults/Cannabinoid Hyperemesis Syndrome

Reevaluation

Reevaluate the patient according to pages 1-3 of this standard operating procedure. Initiate additional diagnostic procedures and treatment if indicated. Does the patients characteristic match criteria?

↓

Corroborate suspicion of CVS/CHS

Check your evaluation for: Major criteria for the diagnosis of CVS and CHS

1. No response to conventional antiemetic and analgesic treatment; 2. Relief of symptoms with hot showering or bathing; 3. Epigastric or periumbilical pain; 4. Polydipsia; 5. Psycho-vegetative symptoms; 6. Average duration of cycles 3 days; 7. Normal eating patterns between episodes; 8. Weight-loss of 5 kg or more; 9. Age below 50 years

↓

Perform esophago-gastro-jejunoscopy, gastric emptying speed analysis and assess for differential diagnoses where indicated: hypercalcemia, chronic inflammatory bowl disease, celiac disease, Addison's disease, psychogenic vomiting, neurologic disorders, abdominal epilepsy, etc. Any organic cause for the symptoms identified?

- **Yes** → Adjust diagnostics and treatment according to your diagnosis; return to SOP if indicated
- **No** → Continue to page 5 of this SOP for further analysis of the case, treatment and differentiation between CVS and CHS where possible.

Standard Operating Procedure:
Detection, evaluation, treatment and follow up of patients possibly suffering from the Cyclic Vomiting Syndrome in adults/Cannabinoid Hyperemesis Syndrome

Differentiation between CVS and CHS

Does the patient meet all essential and at least one major criterion for the diagnosis of CVS and CHS?

No

Reevaluate for differential diagnosis and return to SOP if indicated.

Yes

Check specific criteria indicating CVS or CHS	
Features suggestive for CVS	Features suggestive for CHS
1. Migraine comorbidity; 2. psychiatric comorbidities; 3. rapid gastric emptying; 4. no cannabis abuse	1. Chronic cannabis use*; 2. resolution of symptoms after cannabis cessation; 3. delayed gastric emptying

Follow up (minimum: 12 months)

Evaluate response to cannabis cessation alone

Complete resolution of symptoms: cure

Incomplete response or patient unwilling to cease cannabis

Application of preventive medication, e. g. amitriptyline, propranolol, sumatriptane; application of medication capable to abort an episode within the prodromal phase, e.g. metoclopramide, ondansetron, lorazepam, oxycodone; provide access to physician familiar with CVS and psychosocial care; help identify and avoid triggers, evaluate response to treatment.

*Essential for diagnosis of CHS
